# Deadly scents: Exposure to plant volatiles increases mortality of entomopathogenic nematodes during infection

**DOI:** 10.3389/fphys.2022.978359

**Published:** 2022-09-14

**Authors:** Alexander M. Gaffke, David Shapiro-Ilan, Hans T. Alborn

**Affiliations:** ^1^ Center for Medical, Agricultural and Veterinary Entomology, Agricultural Research Service, United States Department of Agriculture, Gainesville, FL, United States; ^2^ Department of Entomology, Louisiana State University, Baton Rouge, LA, United States; ^3^ Southeastern Fruit and Tree Nut Research Station, Agricultural Research Service, United States Department of Agriculture, Byron, GA, United States

**Keywords:** *steinernema diaprepesi*, heterorhabditis bacteriophora, host immune response, herbivore induced plant volatile (HIPV), below ground biological control

## Abstract

Plants attacked by insects commonly mobilize various defense mechanisms, including the biosynthesis and release of so-called herbivore-induced plant volatiles (HIPVs). Entomopathogenic nematodes (EPNs) can be attracted to these belowground HIPVs, which can enhance biocontrol services from EPNs. However, recent research has also demonstrated that HIPVs can induce and initiate insect immune responses, decreasing the insect’s susceptibility to pathogens and parasites. Therefore, experiments were conducted to test the impact of HIPVs on insects and EPNs during the initial stage of EPN infection. Compounds that can impact EPN attraction and infectivity such as pregeijerene, β-caryophyllene, and α-pinene, and compounds that have been determined to increase or decrease susceptibility of insects to pathogens, such as (*Z*)-3-hexenyl acetate, linalool, and β-ocimene, were selected. Exposure of *Galleria mellonella* larvae to pregeijerene, linalool, β-ocimene and α-pinene during invasion significantly increased mortality of *Steinernema diaprepesi* and *Heterorhabditis bacteriophora* after 48 h. Larval treatment with β-caryophyllene only increased mortality for *Heterorhabditis bacteriophora*. (Z)-3-hexenyl acetate did not cause differential mortality from the controls for either nematode species. In additional experiments, we found that EPNs exposed to α-pinene and linalool were more readily recognized by the insects’ immune cells compared to the control treatment, thus the observed increased mortality was likely due to HIPVs-EPN interactions with the insect’s immune system. These results show that the presence of HIPVs can impact EPN survival in the model host, *G. mellonella*.

## 1 Introduction

Entomopathogenic nematodes (EPNs) are obligate insect parasites in nature that can be effective biocontrol agents for many agricultural pest insects. EPNs of the families Heterorhabditidae and Steinernematidae have only one free living stage, the infective juvenile stage (IJ) (Shapiro-Ilan et al., 2017). The IJs seek out and enter insect hosts via the spiracles, mouth, anus, or intersegmental membranes. While biological control of multiple economically important pests has been successfully achieved with EPNs, multiple cases exist where EPNs fail to control the targeted pest (Georgis et al., 2006).

Recently, significant discoveries and improvements have been made to increase the reliability of EPNs when applied to agroecosystems, such as the use of pheromones and semiochemicals to enhance and regulate the dispersal and infection dynamics of EPNs (Willett et al., 2015; Oliveira-Hofman et al., 2019). For example, it has been demonstrated that exposure of EPNs to root produced terpenoids can result in enhanced movement of EPNs to the source of those terpenoids (Ali et al., 2012; Willett et al., 2015). This movement of the EPNs towards the insect pest also results in increased infection/invasion of the pest by the EPNs (Willett et al., 2015; Willett et al., 2018). These terpenoids are typically produced by the plants in response to insect herbivory, and therefore can be reliable cues of the presence of a host to EPNs. Thus, understanding the belowground interactions of plant volatiles, insects, and entomopathogenic nematodes could be leveraged into better control of root feeding pest insects for agroecosystems.

For EPNs and their symbiotic bacteria, a crucial part of successful insect infection is the tolerance or avoidance of the insect’s immune system (Lewis et al., 2006). Recent research has demonstrated that exposure to plant volatiles can significantly affect the ability of insects to resist infection from microorganisms while other compounds are known to increase the susceptibility of insects to pathogens (Gasmi et al., 2019; Ghosh and Venkatesan et al., 2019). For example, research trials have indicated that exposure of *Spodoptera litura* (F.) to (*E*)-β-Ocimene significantly increased the amount and diversity of immune type hemocytes, which ultimately rendered the insect unattractive to parasitic wasps (Ghosh and Venkatesan et al., 2019). The exposure of *S. litura* to linalool also resulted in significantly higher rates of survival when exposed to *Bacillus thuringiensis* (Bt). In contrast, exposure of *Spodoptera exigua* (Hübner) to linalool increased the susceptibility to a multiple nucleopolyhedrovirus and exposure of *S. exigua* to indole increased the pathogenicity of *B. thuringiensis* (Gasmi et al., 2019). Thus, the same plant produced volatiles that attract EPNs to an insect host might also induce an insect’s defense against invading nematodes. Currently, few investigations have been made to characterize these multitrophic interactions belowground and the impact of nematode attracting chemicals, especially plant produced volatiles, on the behavior and physiology of the target pests is almost completely unknown.

Research has demonstrated that some herbivore induced plant compounds (HIPVs), such as pregeijerene and β-caryophyllene, can be used to enhance the control potential of EPNs in the field (Rasmann et al., 2005; Ali et al., 2012) However, if these compounds increase the resistance of the target pest to EPNs, enhanced biological control may not be achieved and may even be less successful. There is even the possibility that these volatile compounds could negatively impact the biological control services of EPNs in the system to the point where pest pressure actually increases. This paper therefore investigates the impact that exposure to HIPVs could have on the infection rate and success of two EPN species, *Steinernema diaprepesi* Nguyen & Duncan and *Heterorhabditis bacteriophora* Poinar*,* using the model host insect *Galleria mellonella* (L.) (Lepidoptera: Pyralidae). These two genera of EPNs were selected for this study as they represent the two major phylogenies of EPNs and represent different life histories. Additionally, *H. bacteriophora* is one of the most widely used EPN commercially and *Steinernema diaprepesi* has been extensively studied for reaction to volatiles (Ali et al., 2012; Willet et al., 2015; Willet et al. 2017; Willet et al. 2018). Compounds selected for research were based on known importance for entomopathogenic nematode behavior and included pregeijerene, β-caryophyllene, and α-pinene (Rasmann et al., 2005; Ali et al., 2012; Willet et al., 2015). The terpenoids β-ocimene, linalool and the green leafy volatile (Z)-3-hexenyl acetate were selected due to reported priming of insect immune responses (Ghosh and Venkatesan et al., 2019; Gasmi et al., 2019). Investigations of the insect’s immune response to the infecting EPNs were conducted by observation of the cellular response to the nematodes, specifically hemocyte attachment. The cellular response is a primary component of the insect immune system when responding to parasites (Strand 2008).

## 2 Materials and methods

### 2.1 Chemicals

The HIPVs β-caryophyllene, α-pinene, β-ocimene, linalool and (Z)-3-hexenyl acetate were purchased from MilliporeSigma (Burlington, MA, United States). The thermally labile terpenoid pregeijerene was isolated from the root of common rue (*Ruta graveolens L.*) following methods described in Ali et al. (2012). These compounds were selected for testing based on their reported importance to nematode behavior and reports from the literatures of interactions with insect pathogens.

### 2.2 Insects and nematodes

The nematode species *S. diaprepesi* and *H. bacteriophora* were used in all experimentations. Populations of *S. diaprepesi* were collected from citrus groves at the University of Florida Citrus Research and Education Center, Lake Alfred, FL, United States and *H. bacteriophora* HB were provided from the Southeastern Fruit and Tree Nut Research Station, Byron, GA, United States. Nematodes were reared under laboratory conditions at the Center of Medical, Agricultural and Veterinary Entomology, Gainesville, FL, United States. Infective juveniles were propagated using late instars of *Galleria mellonella* obtained from Vanderhorst Wholesale, Inc. (St. Mary’s, OH, United States) (Shapiro-Ilan et al., 2016). Newly emerging IJs from cadavers of *G. mellonella* were collected using White Traps and were stored in 100 ml of deionized water in culture flasks at 14°C for up to 2 weeks until used in research trials. Nematodes were not cleaned or desensitized to residual pheromones from the *in vivo* cultures (Kaplan et al., 2012). Nematodes were not cleaned to maintain the natural exposure and behavioral response of the IJs when they emerge from an insect cadaver. In preliminary trials, it was determined that cleaned IJs failed to disperse and move in experimental trials, resulting in a significant number of no-response trials. In addition, the nematodes were not exposed to any of the chemicals prior to assaying.

### 2.3 Soil column assays

Infection rate and mortality of IJs in *G. mellonella* larvae treated with and without the HIPVs was assessed in 21 mm × 70 mm glass vials filled to a height of 3 cm with wet sand. To set up the experiment, a larva was first placed in the bottom of the vial and covered with sand at field capacity (10% moisture) to a depth of 3 cm. After being covered with sand, a 10 µL syringe was inserted through the sand to the bottom of the vial. The vial was either dosed with 10 µL of pentane, which acted as the control, or 300 ng of either pregeijerene, β-caryophyllene, α-pinene, β-ocimene, linalool or (Z)-3-hexenyl acetate in 10 µL of pentane. Approximately 200 IJs in 200 µL of deionized water was added to the top of the sand column. Once the nematodes were added to the vials, the lids were secured tightly to prevent the loss of the volatile compounds. After 48 h, the trials were stopped, and the larvae were dissected under a microscope and the number of IJs that had invaded were recorded. Infective juveniles that had infected the larvae were categorized as either alive or dead. If the IJs were stiff, crescent shaped and did not respond after probing with a hair bristle, the IJ was classified as dead. If the IJ was moving or responded to stimulus from the bristle, the IJ was recorded as alive (Shapiro-Ilan et al., 2015). This experiment was conducted three times as separate trials with 3-4 replicates per treatment each time the trials were conducted.

### 2.4 Interactions of host hemocytes with entomopathogenic nematodes

*In vitro* studies on the insect immune system and its response to the invading nematodes was conducted from modified methods described in Li et al. (2007) and Manachini et al. (2013). *Galleria mellonella* larvae were placed into the sand column assays as described above and were either treated with 300 ng of a compound in 10 µL of pentane or just 10 µL of pentane to act as a control. Nematodes were also exposed in a similar fashion. One thousand nematodes in 1 ml of di-ionized water were applied to a petri dish (90 mm inner diameter) lined with one sheet of 9 cm diameter Qualitative P5 filter paper (Fisher Scientific, Pittsburgh, PA, United States) and 1 µg of the treatment compounds in 100 µL of pentane or 100 µL of pentane was added as a control. The petri dish was subsequently sealed with parafilm for 48 h. After exposure in the sand column for 48 h, larvae were removed and washed with 70% ethanol in di-ionized water for sterilization. Larvae were held in flexible forceps under slight pressure and induced to bleed through the severing of a foreleg. The drop of hemolymph from the severed foreleg, approximately 10 µL, was subsequently applied onto a glass microscope slide. Two drops of hemolymph were harvested from the same larva, so that paired experiments could be conducted. Individual IJs were collected off the filter paper using a single bristle from a sterilized paintbrush. Nematodes were manipulated into a 3 µL drop of Grace’s insect basal medium (Mediatech, Inc. Manassas, VA, United States). Five control or HIPVs exposed IJs were added to each of the 3 µL drop of Grace’s insect basal medium. Once 5 IJs were added, the suspension was pipetted into the hemolymph from the control and HIPVs exposed larvae. This experiment was replicated twice in time, with 5 replicates of each treatment (HIPVs exposed and control *G. mellonella*, and HIPVs exposed and control IJs). Hemocyte response was recorded at two time points, 30 min and 24 h. The nematodes were observed under a light microscope for hemocytes attachment to the nematodes, and the number of nematodes with hemocytes attached to the cuticle was recorded. Attempts were made to observe hemocyte attachment during the sand column assay, however, conditions of the insects were too deteriorated to positively identify hemocytes during dissection, therefore separate trails were conducted to investigate this interaction.

### 2.5 Statistical analysis

**FIGURE 1:**
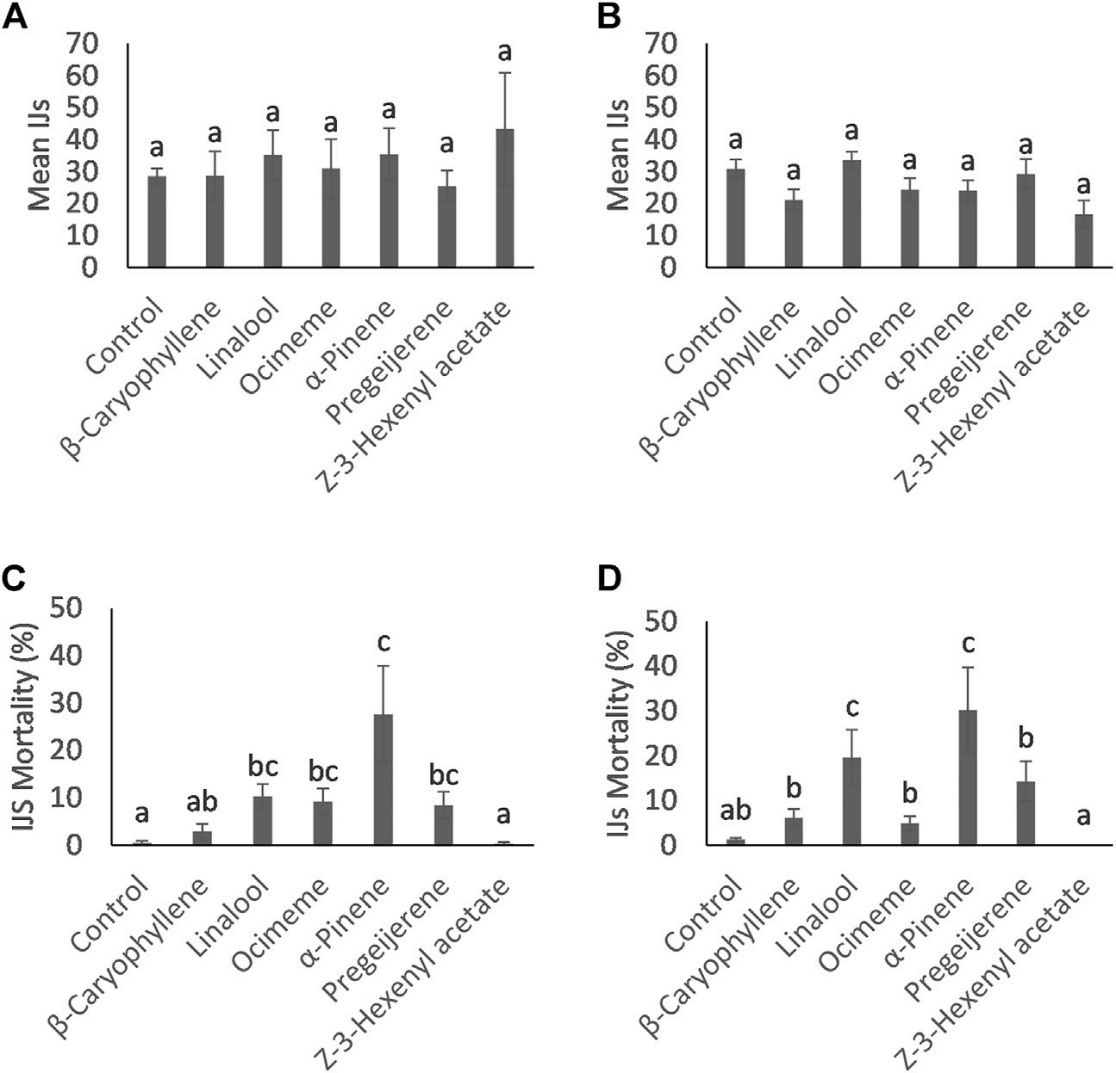
Mean count (±SE) of **(A)** *Steinernema diaprepesi* and **(B)** *Heterorhabditis bacteriophora* Infective Juveniles (IJ) that successfully infected the *Galleria mellonella* host and percent mortality (±SE) of **(C)** *Steinernema diaprepesi* and **(D)** *Heterorhabditis bacteriophora* IJs that infected the *G. mellonella* host. Differing letters above error bars denote statistically significant differences.

The impact of the HIPVs on infection and survival rates of the EPNs were analyzed using analysis of varianceand Tukey’s post hoc analysis. Data was transformed using ln+1 to better match the assumptions of normality and equal variance. Data presented in Figure 1 is of the untransformed data. The response of hemocytes to the entomopathogenic nematodes was analyzed using paired t-tests. Treatments were considered significant if *p* < 0.05. All analysis was conducted using R software version 3.5.1.

## 3 Results

### 3.1 Total infection rates

The total number of IJs for both nematode species that infected the *G. mellonella* larvae during the trials are presented in Figures 1A,B. No compounds increased infection rates of *S. diaprepesi* or *H. bacteriophora* IJs compared to the control (*p =* 0.55 with 6 and 63 days. f.; *p* = 0.07 with 6 and 63 days. f., respectively)*.* All *G. mellonella* larvae were killed by infecting nematodes, indicating that no tested compounds resulted in the loss of ability of the EPNs to kill the insect host.

### 3.2 Mortality rates

**FIGURE 2:**
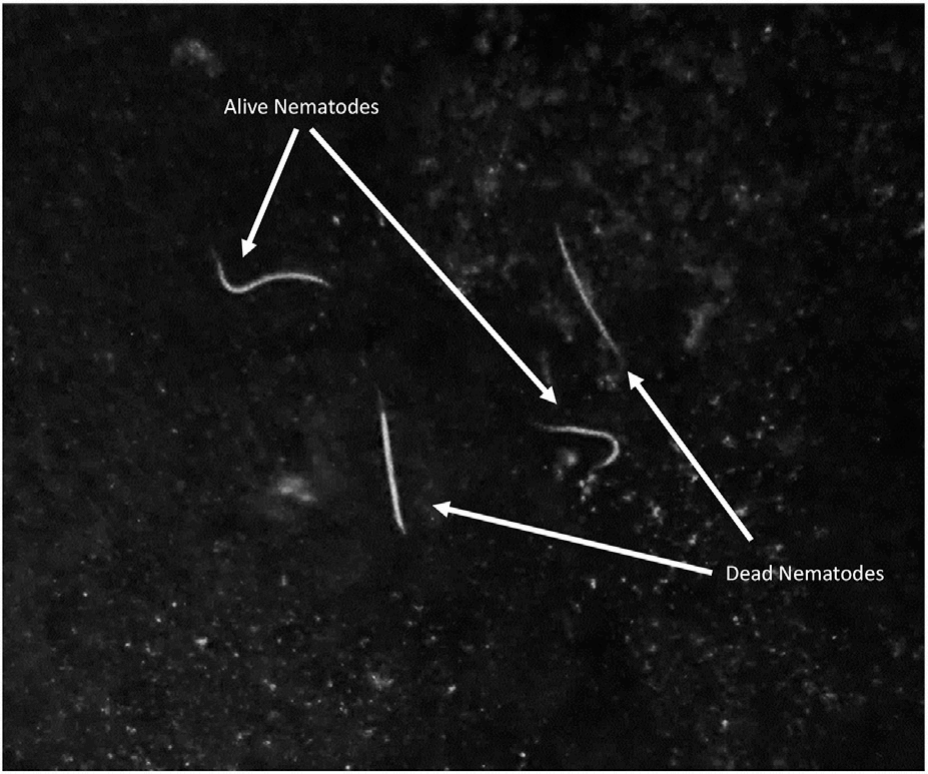
Dead and alive *Steinernema diaprepesi* infective juveniles dissected from a *Galleria mellonella* cadaver exposed to α-pinene.

In contrast to the total number of IJs found in the larvae, the number of dead IJs dissected from the infected *G. mellonella* was significantly different for multiple compounds for both nematode species (Figures 1C,D). Dead IJs were stiff, slightly crescent shaped and did not respond to stimulus (Figure 2). For *S. diaprepesi*, exposure to α-pinene during the infection process resulted in the highest mortality of the IJs (27.6%, *p* = 0.008), followed by linalool, β-ocimene, pregeijerene, β-caryophyllene, and Z-3-hexenyl acetate (10.3%, *p* = 0.009; 9.2%, *p* = 0.003; 8.4%, *p =* 0.004; 2.9%, *p =* 0.15; 0.3%, *p =* 0.7, respectively). Mortality from exposure to β-caryophyllene and Z-3-hexenyl acetate did not differ from the control.

For *H. bacteriophora*, only exposure to α-pinene and linalool during the infection process resulted in significantly higher mortality (34.0% and 24.4%, respectively) (*p* < 0.001 and *p* < 0.001, respectively). Larval mortality was not significantly different with exposure to β-ocimene, pregeijerene, β-caryophyllene and Z-3-hexenyl acetate (*p* = 0.81, *p* = 0.29, *p* = 0.36, *p* = 0.98, respectively).

### 3.3 Hemocyte response

**FIGURE 3:**
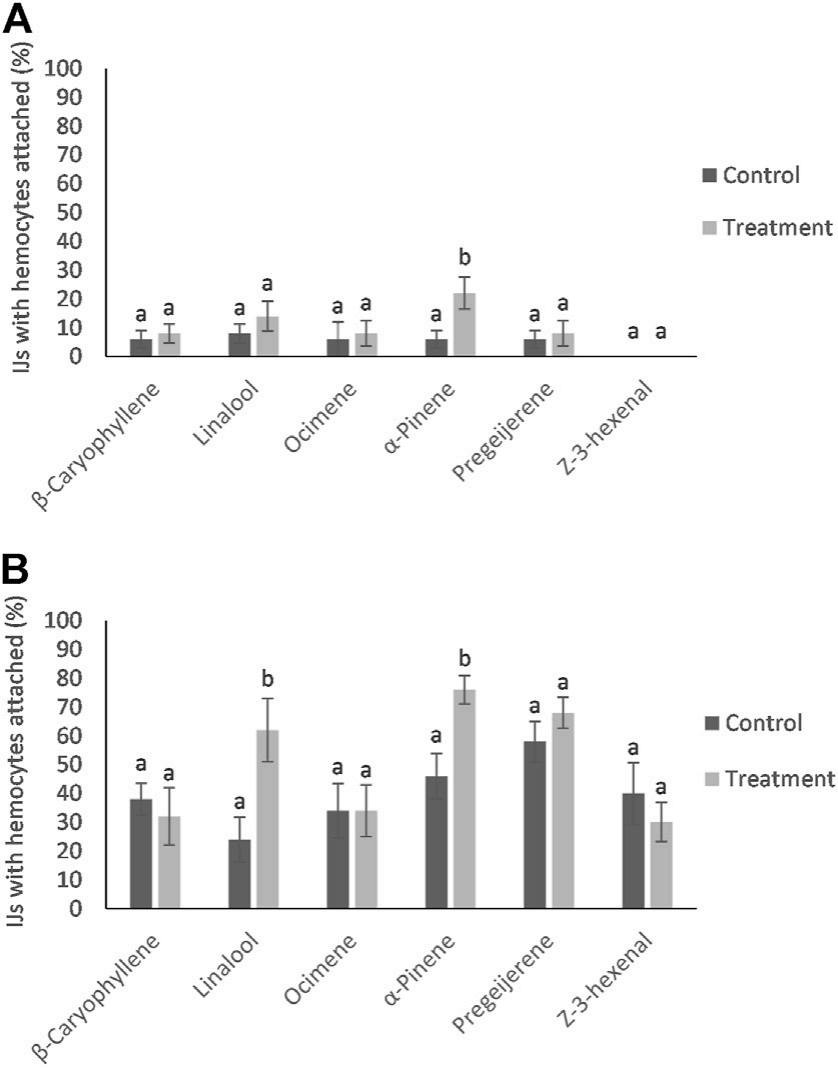
Mean percentage (±SE) of HIPVs exposed Infective Juveniles (IJs) recognized by hemocytes within 30 min for Steinernema diaprepesi **(A)** and Heterorhabditis bacteriophora **(B)**. Different letters above error bars denote statistically significant differences within compounds.

**FIGURE 4:**
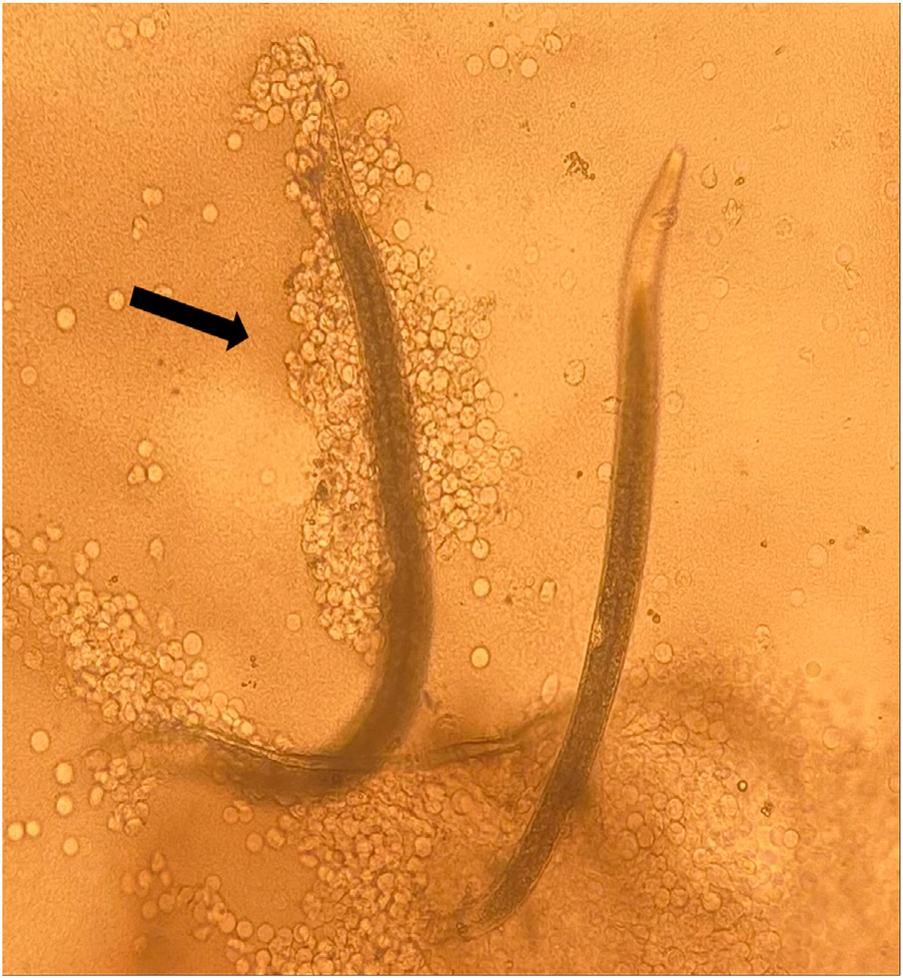
*Steinernema diaprepesi* infective juveniles exposed to α-pinene. Infective Juvenile (IJ) on left immobilized by hemocytes (indicated by arrow) after being placed in insect hemolymph, IJ on right was able to remove attached hemocytes. Immobilized IJ was dead after 24 h s.

No differences were detected in the hemocyte response to the nematodes from control and HIPVs exposed *G. mellonella*. However, when IJs were exposed to the vapor phase of the HIPVs prior to being added to hemolymph, there was a greater chance of hemocyte recognition compared to the control (Figure 3). For *S. diaprepesi*, 23% of IJs exposed to α-pinene for 48 h had hemocytes attach to their cuticle within 30 min compared to 8% for the controls (*p* = 0.01). After 24 h s in the hemolymph, all but three of the *S. diaprepesi* IJs were able to remove the attached hemocytes. The three nematodes that could not remove the hemocytes had very large aggregates of hemocytes attached (greater that 200) and were immobilized (Figure 4). These three nematodes were all from the α-pinene exposed group and died during the 24 h period during which the study was conducted. All other nematodes survived the 24 h period. For *H. bacteriophora* exposed to α-pinene, 76% of IJs had hemocytes attach to their cuticle within 30mins compared to 46% for the control (*p* = 0.04). For *H. bacteriophora* exposed to linalool, 62% of the IJs had hemocytes attach to their cuticle within 30 min compared to 24% for the control (*p* = 0.03). After 24 h s, all *H. bacteriophora* IJs were able to remove the attached hemocytes, mainly through the shedding of the second instar cuticle that the IJs retain before infection. All *H. bacteriophora* survived the 24 h period of this study. *Heterorhabditis bacteriophora* had a greater likelihood and greater variability of hemocyte attachment compared to *S. diaprepesi*.

## 4 Discussion

While our understanding of HIPVs in the soil and the belowground interactions they govern is limited, ongoing research is providing evidence that these belowground interactions can influence EPNs and herbivores (Tonelli et al., 2016; Grunseich et al., 2020). Herbivore induced plant volatiles play a critical role in regulating pest densities in agriculture and have also been shown to recruit and increase the biological control services of EPNs in agroecosystems (Hassani-Kakhki et al., 2020). This study indicates that HIPVs may also have antagonistic interactions with EPNs. The discovery that HIPVs compounds could reduce the efficacy of EPNs is of great significance. The mortality of 28% and 34% of the EPNs exposed to α-pinene invading a highly susceptible host in a highly controlled laboratory environment could translate to complete failure of the EPNs to kill pest insects in an agroecosystem, especially when other biotic and abiotic factors are acting on the EPNs. Entomopathogenic nematodes commonly utilize “mass attack” to overcome host defenses, and the mortality of that many IJs upon invasion could prove significant (Peter and Ehlers 1997; Lewis et al., 2006).

It should be noted in this study no *G. mellonella* larvae fully escaped infection during the bioassays, and all assayed larvae were killed by the EPNs. This suggests for this host that the biocontrol services of the EPNs were not lost. However, these results indicate impacts on the potential for the EPNs to recycle and produce the next generation. The importance of nematodes recycling has been observed when attempting to control the pecan weevil, *Curculio caryae* (Horn) (Coleoptera: Curculionidae). Long term recycling of nematodes in *C. caryae* was unlikely due to reduced reproduction in that insect host, requiring retreatment of the nematodes (Shapiro-Ilan et al., 2005). If the goal of an EPN application is for them to persist in the environment and provide long term control, the IJ morality from the HIPVs is more likely to be detrimental, as these IJs would not be able to reproduce and replace their numbers. Given that HIPVs attract more IJs and cause higher infection rates, the increased attraction and infection may outweigh the negative effects of the mortality from HIPV exposure. Field tests or multigenerational rearing experiments with various hosts and nematode species, would be needed to determine if this observed interaction is positive or negative for long term biocontrol with EPNs.

Nematodes are well documented in the literature to survive exposure to these HIPVs (Willett et al., 2015; Willet et al., 2017; Willet et al., 2018), and in this investigation no mortality of the IJs was observed after direct exposure of the IJs to the HIPVs It is likely that the mortality of the IJs was not arising from nematocidal properties of the HIPVs. Therefore, the increased mortality of the IJs was likely arising from interactions with the insect’s immune system. The increased incidence of hemocyte attachment within the first 30 min of EPNs encountering hemolymph provides a possible explanation for the increased mortality and supports the hypothesis that the insect is killing the IJs when they enter the body. While conducting the studies, discoloring/darkening of the dead nematodes was not noticed, suggesting that the mortality is not being achieved through melanization of the IJs, as melanized IJs are usually indicated by brown colorations (Li et al., 2007). This observation provides further evidence the increased level of mortality is being driven by the cellular immune response to the nematodes. It was initially hypothesized that the HIPVs would primarily be working on the insect’s immune system, as reported by Gasmi et al. (2019) and Ghosh and Venkatesan et al. (2019), and not interacting with the EPNs. Surprisingly, the hemolymph from the larvae exposed to HIPVs did not have a detectable impact on the EPNs, only when the EPNs where exposed to the HIPVs did hemocyte attachment increase.

Why the hemocytes are better able to recognize and attach to the nematodes after HIPV exposure is not known and will need to be further investigated to determine the mechanism resulting in this observation. However, it is well documented that one of the mechanisms EPNs use to evade the insect immune response is surface coat proteins. It could be hypothesized that the presence of the terpenoids in the vapor phase prior to the nematodes infecting the larvae is resulting in changes to the surface coat proteins or other excreted/secreted products, resulting in reduced ability of the EPNs to avoid the insect immune system. Terpenes have been demonstrated to interact with proteins, changing linkages and properties of the proteins (Gouda et al., 2018). The difference in surface coat proteins between the two nematode species may also explain the variability in mortality and hemocyte attachment between the two species.

In addition to further research on HIPVs-herbivore-nematode interactions, research also needs to be conducted on the basic chemistry and interaction of the HIPVs to the soil environment. For example, recent research has identified that linalool breaks down into α and β-pinene in the soil environment (Som et al., 2017). In our current trials, linalool contributed to moderate levels of mortality to the infecting IJs, but α-pinene resulted in the highest levels of mortality for both species. Many *Solanaceae* species release linalool in the soil (Būda and ČepulytĖ-RakauskienĖ 2011). The breakdown of linalool to α and β pinene, and the subsequent interaction between an EPN and the insect’s immune system may decrease the ability of EPNs to control root pests in *Solanaceae* production. Thus, assaying of volatiles released by cleaned exposed roots rather than their potential in-soil break down products may result in improper conclusions. A similar scenario has already been demonstrated with EPN-plant-insect interactions (Ali et al., 2012). It was initially concluded that EPNs were attracted to the HIPV geijerene, while it was later determined that the actual compound produce by the plant was pregeijerene and the identification of geijerene was an artifact from the chemical analysis. Other common HIPVs found in the rhizosphere of agroecosystems should be investigated for their interactions between EPNs and insect immune systems. The exploration of these interactions will progress the scientific communities understanding of the variables that increase or hinder the biological control potential of EPNs in agroecosystems. For example, commercial blueberry fields are commonly mulched with pine bark mulch which will contain a high concentration of terpenoids (Clark and Moore 1991). If these compounds decrease the susceptibility of the pests to EPNs in a similar fashion as *G. mellonella*, this might explain why EPN diversity in blueberry fields are reduced (Rivera et al., 2016). The recommendation could then be made to mulch with alternative sources that would not introduce compounds that decrease the efficacy of EPNs.

The symbiotic bacteria carried by EPNs are also known to suppress the immune response, but the impact of the bacteria is believed to be later in the infection process, 30 min-5 h post infection, and not within the early-time frame of this study (Ciche and Ensign 2003). During this study, the hemocytes had a propensity to attach to the mouth and head of the EPNs. This observation is curious, as some EPNs are known to expel their symbiotic bacteria through the mouth (i.e., *Heterorhabditis bacteriophora*) while other species [*S. carpocapsae* (Weiser)] defecate the bacteria (Ciche et al., 2006). It could be hypothesized that attachment of the hemocytes to the mouth could prevent or slow the releases of regurgitated bacteria. Other studies have also identified a high propensity of the hemocytes to attach to the anus (Li et al., 2007). In most instances, this study only identified a modest number of hemocytes attaching to the nematodes, typically less than 20, however, in an intact insect the recognition and response of the hemocytes could continue to build and result in a cascade of events that would result in the encapsulation of the nematode.

This paper focused on the impacts of the HIPVs on the EPNs, but EPNs also carry with them symbiotic bacteria that are critically important for infection and survival of the IJs in the insect host. Therefore, the potential regulation of the insect immune system could also be acting on the symbiotic bacteria once they are released into the hemolymph, thus, further studies will be needed involving the symbiotic bacteria. Moreover, additional research will be needed to further explore our findings, such as investigations into the mechanism behind the increased IJ mortality, dose response to the HIPVs, and if blends of HIPVs interact differently with the EPNs. The evolutionary context for these negative interactions also needs to be investigated, as HIPVs have traditionally been considered beneficially to trophic levels above the consumer. These result may indicate that consumers are hijacking the plants response to try and protect themselves. Additionally, other EPN-host systems should be explored, and the impact of HIPVs on EPN-host interactions must be further explored under field conditions.

## Data Availability

The raw data supporting the conclusion of this article will be made available by the authors, without undue reservation.
